# Recording Patient Data in Burn Unit Logbooks in Rwanda: Who and What Are We Missing?

**DOI:** 10.1093/jbcr/iraa198

**Published:** 2020-10-31

**Authors:** Elizabeth Miranda, Lotta Velin, Faustin Ntirenganya, Robert Riviello, Francoise Mukagaju, Ian Shyaka, Yves Nezerwa, Laura Pompermaier

**Affiliations:** 1 Program in Global Surgery and Social Change, Harvard Medical School, Boston, MA; 2 Division of Vascular Surgery, University of Southern California, Los Angeles, CA; 3 Surgery and Public Health, Department of Clinical Sciences Lund, Faculty of Medicine, Lund University, Lund, Sweden; 4 Plastic Surgery Unit, Department of Surgery, University Teaching Hospital Kigali (CHUK), Kigali, Rwanda; 5 Center for Surgery and Public Health, Brigham and Women’s Hospital, Boston, MA; 6 Plastic Surgery Department, Rwanda Military Hospital, Kigali, Rwanda; 7 Linköping University Hospital, Linköping, Sweden

## Abstract

Systematic data collection in high-income countries has demonstrated a decreasing burn morbidity and mortality, whereas lack of data from low- and middle-income countries hinders a global overview of burn epidemiology. In low- and middle-income countries, dedicated burn registries are few. Instead, burn data are often recorded in logbooks or as one variable in trauma registries, where incomplete or inconsistently recorded information is a known challenge. The University Teaching Hospital of Kigali hosts the only dedicated burn unit in Rwanda and has collected data on patients admitted for acute burn care in logbooks since 2005. This study aimed to assess the data registered between January 2005 and December 2019, to evaluate the extent of missing data, and to identify possible factors associated with “missingness.” All data were analyzed using descriptive statistics, Fisher’s exact test, and Wilcoxon Rank Sum test. In this study, 1093 acute burn patients were included and 64.2% of them had incomplete data. Data completeness improved significantly over time. The most commonly missing variables were whether the patient was referred from another facility and information regarding whether any surgical intervention was performed. Missing data on burn mechanism, burn degree, and surgical treatment were associated with in-hospital mortality. In conclusion, missing data is frequent for acute burn patients in Rwanda, although improvements have been seen over time. As Rwanda and other low- and middle-income countries strive to improve burn care, ensuring data completeness will be essential for the ability to accurately assess the quality of care, and hence improve it.

## INTRODUCTION

The systematic data collection in burn centers of high income countries (HICs) demonstrates a decreasing incidence, mortality, and morbidity after burn injuries in recent decades, whereas the lack of, or incompleteness of, information from low- and middle-income countries (LMICs) hinders an accurate global overview of burn epidemiology.^1^ In HICs, burn patients’ data are commonly registered in dedicated, electronic burn registries.^2^ In LMICs, data on burn injuries are more frequently recorded in logbooks or as a part of a trauma registry.^3,4^ The implementation of trauma registries has been associated with better patient outcomes, however, incompleteness and poor quality of data have been reported as challenges in LMICs, in particular relating to mortality.^3,4^ Although missing data may hide information that could improve patient outcomes and guide effective healthcare strategies, only few studies, to the best of our knowledge, have looked at the “missingness” of data collected in trauma patients and no study exists on burn patients specifically.^5,6^

Rwanda is a country in East Africa, where trauma has been estimated to account for 22% of deaths in the capital city, Kigali.^7^ Between 2011 and 2016, data on trauma patients at the University Teaching Hospital of Kigali (CHUK) were collected in the pilot-project “Rwanda Injury Registry” (RIR).^8^ Burns were found to be among the most common injury mechanisms, and together with road accidents, the most lethal. However, the purpose of the RIR was not burn-specific and therefore data relevant to understand burn epidemiology, such as burn mechanism, characteristics, or surgical treatment provided, were not reported. CHUK is home to the only designated burn unit in Rwanda and since 2005, the staff at the CHUK burn ward have collected data about the admitted burn patients in logbooks.

This study aims to explore the data collected in the CHUK burn ward logbooks between 2005 and 2019, trying to highlight and explain possible and repetitive trends in missing data. Results of this analysis will be useful to develop a burn database specific for the need in a low- and middle-income setting.

## METHODS

### Study Location

This study was conducted in the burn unit at the University Teaching Hospital of Kigali (CHUK), Rwanda, which admits patients with acute burn injuries and is currently the only unit designated for burn care in Rwanda. The burn unit is staffed by bedside nurses, has one attending plastic surgeon, one plastic surgery resident (of the total three in the country) rotate at CHUK at a time, one general surgery resident, one physiotherapist, one psychologist, one nutritionist, and a social worker. In the Rwandan healthcare system, the majority of patients who need specialized care must be referred to tertiary centers from their local health center or district hospital. CHUK is the main referral hospital, receiving up to 75% of all surgical referrals of the country, including but not limited to patients from Kigali, the Northern, and Western provinces. It also receives some of the most critical patients from the Southern and Eastern provinces along with Rwanda Military Hospital.

### Data Collection and Analysis

**Figure 1.**
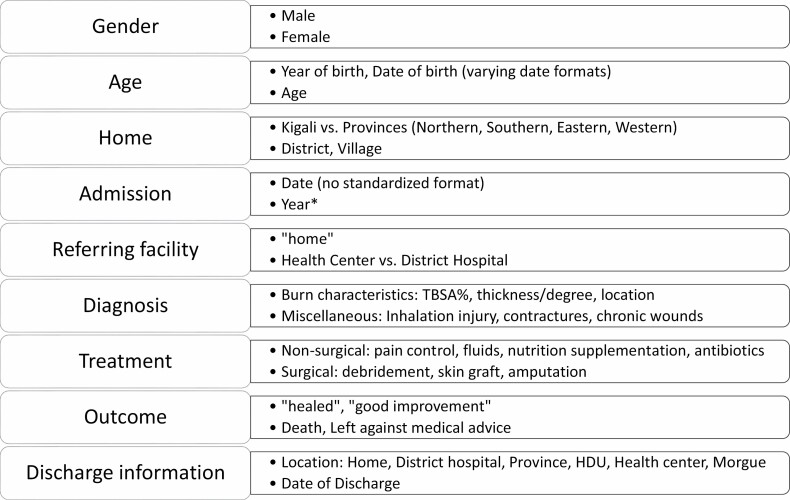
Data currently collected in CHUK burn ward logbooks. Variables on the left are the data categories listed in the logbooks. The right column shows examples of information recorded under each variable. *HDU*, high dependency unit; *TBSA*, Total Burn Surface Area.

The study was approved by the CHUK Ethics Review Committee (Ref:EC/CHUK/007/2020) and Partners IRB Committee (Ref:2020P001096). Data on patients admitted at CHUK burn wards for treatment of acute burns has been systematically collected by burn ward nurses in logbooks since 2005 (Figure 1). In this study, we included following data collected between January 2005 and December 2019: age, gender, province of origin, date of admission, referral pathway (from district hospital, health center or the patients’ home), burn mechanism, presence of full thickness burns, total body surface area burnt percentage (TBSA%), surgical treatment, date of discharge, and in-hospital mortality.

Patients were considered to have missing burn thickness data if they had neither burn thickness nor burn degree recorded in the logbook.

Missing mortality data was calculated as patients who were neither recorded as “dead” nor “healed”/”discharged”/”left against medical advice.” If one or more variables were missing, the record was considered incomplete. All available, de-identified data was retrospectively put into an Excel spreadsheet in December 2019. Statistical significance (considered at *P*-value ≤.05) in the correlation of various factors and the presence of missing data was calculated using Fisher’s exact test. Wilcoxon rank-sum test was used to test for differences in medians of continuous variables. Stata IC 16.0 (Software7) was used for statistical analysis.

## RESULTS

Between January 2005 and December 2019, 1093 records of acute burn injury were recorded in the CHUK burn unit logbook. Based on the available data, the median age of the patients was 3.4 years (IQR 1.8–16.5), the median TBSA 17.0% (IQR 12–25%), and 44.4% of them were female (*n* = 476/1073). The most common type of burn injury was scalds (*n* = 639/789, 81.0%), followed by flame injuries (*n* = 121/789, 15.4%), and the in-hospital mortality was 13.0% (*n* = 121/931).

Of the 1093 records, 702 (64.2%) had incomplete data and the only variable reported in all patient records was the year of admission (Table 1). After this, the most commonly reported variables were gender (*n* = 1073/1093, 98.2%), province (*n* = 1064/1093, 97.4%), and age (*n* = 1052/1093, 96.3%). The most frequently missing data variables were whether surgical intervention was performed (*n* = 381/1093, 34.9%), and the referring facility (home vs health center vs district hospital; *n* = 432/1093, 39.5%). Only four patients were recorded as being referred from a health center and all of these patients had incomplete data.

**Table 1.** Comparison of complete data with incomplete data, 2005–2019

	All Patients (*n* = 1093)	Complete Data* (*n* = 391)	Incomplete Data^†^ (*n* = 702)	*P*
Year admitted				<.001**
2005–2010	310 (28.4)	10 (2.6)	300 (42.7)	
2011–2015	334 (30.6)	43 (11)	291 (41.5)	
2016–2019	449 (41.1)	338 (86.5)	111 (15.8)	
Gender				.848
Male	597 (55.6)	216 (55.2)	381 (55.9)	
Female	476 (44.4)	175 (44.5)	301 (44.1)	
Age groups, years				.793
0–5	643 (61.1)	237 (60.6)	406 (61.4)	
6–15	149 (14.2)	57 (14.6)	92 (13.9)	
16–39	203 (19.3)	73 (18.7)	130 (19.7)	
40–60	39 (3.7)	18 (4.6)	21 (3.2)	
>60	18 (1.7)	6 (1.53)	12 (1.8)	
Province				.088
Kigali	735 (69.1)	258 (66.0)	477 (70.9)	
Outside Kigali	329 (30.9)	133 (34.0)	196 (29.1)	
Referring facility				*.012
Home	245 (37.8)	156 (39.9)	93 (34.7)	
Health Center	4 (0.6)	0 (0)	4 (1.6)	
District Hospital	410 (62.2)	235 (60.1)	175 (65.3)	
Burn thickness				.587
Full	199 (38.9)	100 (37.7)	99 (40.2)	
Partial	312 (61.1)	165 (62.3)	147 (59.8)	
Burn degree				.159
First	7 (0.97)	0 (0)	7 (1.4)	
Second	663 (92.2)	209 (94.1)	454 (91.4)	
Third	49 (6.8)	13 (5.9))	36 (7.2)	
Burn mechanism				.415
Scald	639 (81.0)	312 (79.8)	327 (82.2)	
Other	150 (19.0)	79 (20.2)	71 (17.8)	
Burn treatment				.002**
Medical management only	600 (84.3)	345 (88.2)	255 (79.4)	
Surgery	112 (15.7)	46 (11.8)	66 (20.6)	
Discharged to lower level of care	804 (86.4)	346 (88.5)	458 (84.8)	.122
In-hospital mortality	121 (13.0)	44 (11.3)	77 (14.3)	.200

Bold values indicate statistical significance.

* Data are presented in numbers (%) of patients with complete data recorded.

^†^Data are presented in numbers (%) of patients with incomplete data.

The strongest predictor of completeness of recorded data was the year in which the patient presented to the burn ward (*P* < .001), and, in general, data became more complete over time (Table 2). The exceptions to this were gender, which was equally reported over time, and in-hospital mortality, the accuracy of which does not seem to follow any trend over the years. No significant difference in completeness of recorded data was seen between different age groups. When comparing completeness of data between those who died during admission and those discharged, those who died were more likely to be missing information regarding burn mechanism (*P* = .008), degree (*P* = .01), and whether the patient required surgical intervention (*P* = .014) (Table 3).

**Table 2.** Comparison of incomplete data over time

	2005–2010* (*n* = 300)	2011–2015* (*n* = 291)	2016–2019* (*n* = 111)	*P*
Age	9 (2.9)	24 (7.2)	8 (1.8)	<.001**
Gender	7 (2.3)	6 (1.8)	7 (1.6)	.779
Referring location (Kigali vs province)	17 (5.5)	5 (1.5)	7 (1.6)	.003**
Referring facility (home, health center, district hospital)	230 (74.2)	197 (59.0)	5 (1.1)	<.001**
Date of admission	8 (2.6)	65 (19.5)	8 (1.8)	<.001**
Burn mechanism	211 (68.1)	88 (26.4)	5 (1.1)	<.001**
Burn thickness	181 (58.4)	281 (84.1)	120 (26.7)	<.001**
Burn degree	76 (24.5)	64 (19.2)	234 (52.1)	<.001**
TBSA %	228 (73.6)	116 (34.7)	23 (5.1)	<.001**
Treatment	230 (74.2)	147 (44.0)	4 (0.9)	<.001**
Date of discharge	28 (9.0)	64 (19.2)	33 (7.4)	<.001**
In-hospital mortality	86 (27.7)	19 (5.7)	57 (12.7)	<.001**

*TBSA*, Total Burn Surface Area. Bold values indicate statistical significance.

*Data are presented in numbers (%) of patients with incomplete data on the specified variable.

**Table 3.** Comparison of incomplete data between patients who died during admission and those who survived to discharge

	In-Hospital Deaths* (*n* = 121)	Discharged alive^†^ (*n* = 810)	*P*
Age	4 (3.3)	22 (2.7)	.765
Gender	1 (0.8)	4 (0.5)	.502
Referring location (Kigali vs province)	2 (1.7)	5 (0.6)	.228
Referring facility (home, health center, district hospital)	51 (42.2)	277 (34.2)	.102
Date of admission	13 (10.7)	52 (6.4)	.086
Burn mechanism	41 (33.9)	182 (22.5)	.008**
Burn thickness	58 (47.9)	413 (51.0)	.559
Burn degree	54 (44.6)	264 (32.6)	.010*
TBSA%	44 (36.4)	246 (30.4)	.207
Treatment	48 (39.7)	231 (28.6)	.014*

*TBSA*, Total Burn Surface Area.

*Data are presented in numbers (%) of patients who died in-hospital with incomplete data on the specified variable.

^†^Data are presented in numbers (%) of patients who were discharged alive with incomplete data on the specified variable.

**Figure 2.**
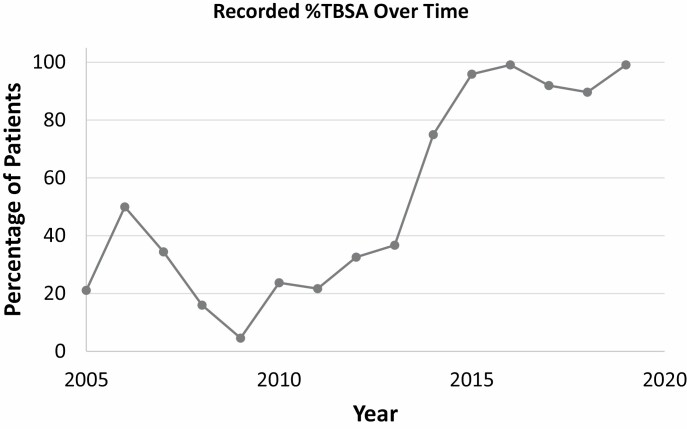
Trend over time of percentage of patients with recorded TBSA%.

The trend in reporting data on burn characteristics (burn thickness, burn degree, and TBSA%) changed significantly over time. Burn degree was less consistently recorded at the end of the study period (24.5% incomplete from 2005 to 2010 vs 52.1% incomplete from 2016 to 2019, *P* < .001), while, on the contrary, burn thickness and TBSA% were more (58.4% incomplete thickness data from 2005 to 2010 vs 26.7% incomplete from 2016 to 2019, and 73.6% incomplete TBSA% data from 2005 to 2010 vs 5.1% incomplete from 2016 to 2019, *P* < .001) (Figure 2). Incomplete burn degree data was more frequent in female than in male patients (39.1% vs 28.8%, *P* < .001). Patients referred from Kigali were more likely to be missing thickness information as compared to burn degree (30.9% vs 38.0%, *P* = .024). Those referred from home or from health centers were more likely to be missing burn thickness information than those referred from district hospitals (51.8% vs 28.5%, *P* < .001), while those from district hospitals were missing more TBSA% data than those from home or health centers (27.6% vs 19.7%, *P* = .025). Patients with missing burn thickness information had smaller TBSA% than those with recorded thickness information (median 15.0% vs 18.0%, *P* = .006). However, no significant difference in burn extension was seen between those with complete and incomplete data (median TBSA 16.0 and 18.0%, respectively, *P* = .15). Patients who had burns caused by other than scald injuries were more likely to have missing data on thickness (56.0% vs 42.9%, *P* = .005) and on burn degree (40.7% vs 30.5%, *P* = .02) as compared to those with scald burns.

Associations were also seen between patient characteristics and surgical treatment information. Those patients whose injuries were managed without surgery were more likely to be missing information on burn thickness or burn degree than those who underwent surgical intervention (missing data on burn thickness in patients without surgery vs those with surgery = 46.0% vs 17.9%, *P* < .01; on burn degree = 35.8% vs 23.2%, *P* = .02). However, patients with partial thickness burns were more likely to have incomplete information on the surgical care delivery than those with full thickness burns (25.6% vs 7.5%, *P* < .001). Patients referred from within Kigali were also more likely to have incomplete treatment information during the admission at CHUK as compared to those referred from outside of Kigali (35.8% vs 28.0%, *P* = .014). Finally, the missing data on the treatment provided at the CHUK declined significantly during the study period (from 74.2% between 2005 and 2010, to 44.0% in 2011–2015, to 0.9% in 2016–2019, *P* < .001).

## DISCUSSION

Burns are a major cause of long-term morbidity and mortality in sub-Saharan Africa.^1,9,10^ The analysis of burn patient epidemiology and of outcome after provided treatment is essential to assess and improve quality of burn care. To perform an accurate analysis, it is necessary to know which data are available and which data are missing.^11^ The main goal of this retrospective study was to analyze the data collected in the logbooks of the CHUK burn ward, between January 2005 and December 2019, in an attempt to find a recurrent pattern that could explain the reasons for data incompleteness. To the best of our knowledge, this is the first study to assess missingness of data in a burn registry in an LMIC.

Of the 1093 burn patients recorded during the study period, just 36% had complete data. Demographic data, such as age, gender, and province of origin of the patients, were those most consistently collected, while information regarding the referring facility and treatment were frequently missing. These findings suggest that registration of data is a part of standard care, whereas there was no consistent strategy to ensure continued recording of data during the hospitalization. In fact, demographic data is retrievable from patient documents and does not require extra time to interrogate the patients (or their relatives) or to follow them during the time at the ward. Our study aligns with the current literature on missing data in trauma registries which reports high levels of missingness in clinical and outcomes variables, and higher completeness in collection of demographic variables like age or gender.^6,12^

Previous studies report that severity of condition and mortality is associated with missing data^3,5^ and this study could confirm this association. In fact, burn mechanism, burn degree, and need for surgical intervention were more likely to be missing in the records of patients with in-hospital mortality than patients who were alive at discharge. A possible explanation of this finding is that critical patients often are transferred to the intensive care unit (ICU) where they eventually die, however, the logbooks only report data collected at the burn ward, by burn ward nurses. To obtain complete information, it would be necessary to implement a burn data registry that follows the patients throughout the hospital stay, from admission to the end; to facilitate this process, an online database accessible from each department would be ideal.

The assessment of burn severity (TBSA%, presence of full-thickness burns, and burn degree) requires specific competence, which may be facilitated by the presence of a physician with burn care expertise who evaluates the patient.^13^ Furthermore, to facilitate the registration of these data, clear communication between the physician who estimates the burn and the nurse who records the data is essential. However, reporting of burn degree did not improve over time which could be due to this largely being replaced by burn thickness as a way to express burn severity.^14^

This study indicates that the incompleteness of data collection is the consequence of systematic errors, rather than of random ones. In fact, missing data could be attributed to poor or lacking communication, between physicians and nurses or between the burn ward and the ICU. The high prevalence of missing data may be caused by the lack of a routine and standardized system of data collection and this could be implemented by assigning to a staff member the responsibility to periodically check the data collection process. Probably, a high workload at the burn ward may also contribute to data incompleteness, which in turn could be attributed to the low physician-density. In 2014, CHUK received its first plastic surgeon, which treats both burn patients and general plastic surgery patients. In 2019, the first Rwandan plastic surgery residents started rotating at the ward, which had previously been covered by general surgery residents who would rotate in plastic surgery for 3 months. While these two time points are correlated with a positive inflection in data completeness, the completeness of data collection also improved significantly over the time in between the points.

**Figure 3.**
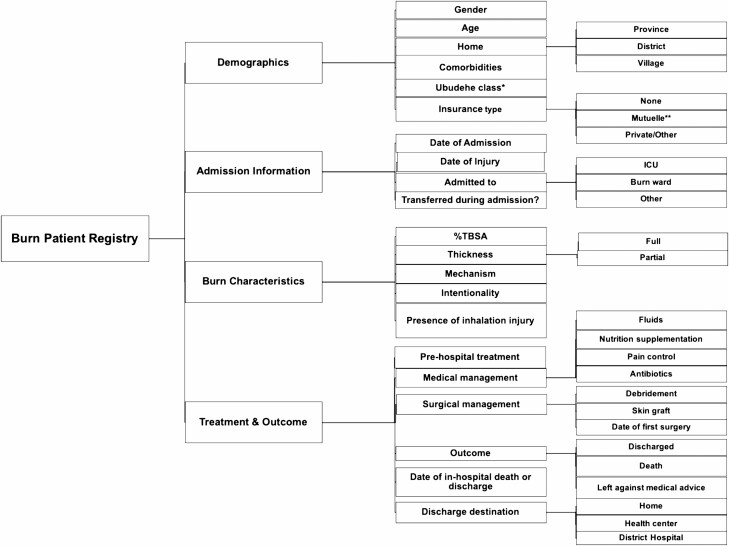
Recommended variables to be collected in a Rwandan burn registry. *Ubudehe class is a Rwandan socioeconomic classification system based on income, where 1 is the lowest level and 4 is the highest.^15^ **Mutuelle, a community-based insurance, is the most common insurance type in Rwanda.^16^*ICU*, intensive care unit.

To address the issue of missing data, we recommend the implementation of a burn registry with contextually relevant variables to standardize data recording (Figure 3). An ideal template to standardize information collection for burn patients at CHUK is the World Health Organization’s Global Burn Registry (GBR). The GBR was launched in 2018 to provide a standardized framework for recording data regarding burn patients. It was developed in conjunction with burn experts from around the world and piloted in multiple countries prior to its release. A review of the GBR in 2019 showed that low-income countries are currently underrepresented—given this, it would benefit from the participation of Rwanda’s referral centers.^17^ However, we would recommend a modified version of the GBR, with questions that capture information relevant to the local context as well as information about surgical treatment that the patient receives. In our list of suggested variables for a Rwandan burn unit registry, we recommend the addition of variables such as Ubudehe class (Rwandan socioeconomic classification system), education level, insurance type, admission location, use of pre-hospital treatment, comorbidities, injury intentionality, presence of inhalation injury, burn location on the body, and admission to ICU. We believe that contextualization of the data collection would improve understanding of the collection process among staff, increasing its compliance. However, first, it would be useful to conduct a qualitative study with burn ward nurses to explore in-depth the causes of missing data collection. Secondly, it would be important to assess with a pilot study whether implementation of a burn unit registry such as GBR would impact data completeness.

This study is not without limitations. As a retrospective study, it is limited by the quality of the data that was collected. No data variables regarding prehospital treatment were collected, preventing a holistic understanding of epidemiology from the time of injury. There may be critical burn patients that were only treated in the ICU and therefore not recorded in the burn unit logbook. Additionally, there was a significant amount of variation in how data was recorded, and in some cases, assumptions needed to be made to interpret this information. For example, when looking at the referring facility variable, only four patients were recorded as referred from their local health center, whereas 245 was recorded as coming directly from home. According to the structure of the Rwandan healthcare system, patients are intended to seek care at their local health center and only seek care directly at a higher level in exceptional cases. Therefore, it is unlikely that so many patients seek care directly at the highest specialized tertiary center of the country and not at the local health centers first, especially for those patients living outside of Kigali. This highlights another important limitation is that this study only assesses data completeness; not accuracy or validity of data.^14^

## CONCLUSION

Missing data is highly prevalent among burn patients treated at a tertiary hospital in Rwanda, although significant improvement in data completeness has been seen in the past 5 years. Incomplete reporting of data is associated with in-hospital mortality, indicating that missing data is the consequence of systematic errors, rather than random. As the first study of missing data in a burn registry in an LMIC, this study may aid Rwanda and other LMICs to ensure data completeness in the drive to improve burn care.
